# Manifold epigenetics: A conceptual model that guides engineering strategies to improve whole-body regenerative health

**DOI:** 10.3389/fcell.2023.1122422

**Published:** 2023-02-14

**Authors:** Choong Yong Ung, Cristina Correia, Daniel Denis Billadeau, Shizhen Zhu, Hu Li

**Affiliations:** ^1^ Department of Molecular Pharmacology and Experimental Therapeutics, Mayo Clinic, Rochester, MN, United States; ^2^ Department of Immunology, Mayo Clinic, Rochester, MN, United States; ^3^ Department of Biochemistry and Molecular Biology, Mayo Clinic, Rochester, MN, United States

**Keywords:** regenerative health, aging, manifold epigenetics, epigenetics memory, body-wide phenotypes, epigenetics

## Abstract

Despite the promising advances in regenerative medicine, there is a critical need for improved therapies. For example, delaying aging and improving healthspan is an imminent societal challenge. Our ability to identify biological cues as well as communications between cells and organs are keys to enhance regenerative health and improve patient care. Epigenetics represents one of the major biological mechanisms involving in tissue regeneration, and therefore can be viewed as a systemic (body-wide) control. However, how epigenetic regulations concertedly lead to the development of biological memories at the whole-body level remains unclear. Here, we review the evolving definitions of epigenetics and identify missing links. We then propose our Manifold Epigenetic Model (MEMo) as a conceptual framework to explain how epigenetic memory arises and discuss what strategies can be applied to manipulate the body-wide memory. In summary we provide a conceptual roadmap for the development of new engineering approaches to improve regenerative health.

## Introduction

Regenerative health is based on the idea that we can utilize the body’s own regenerative mechanisms to restore or regenerate tissues to promote better health. Regenerative medicine is an emerging field, and strategies like tissue engineering (Hofer and Lutolf, 2021), development of biomaterials (Gilbert et al., 2021), medical devices (Kim et al., 2019) and artificial organs (Tetta et al., 2003), as well as cellular therapies (Culme-Seymour et al., 2012) are currently paving new opportunities to improve the overall quality of life for patients. In particular, breakthrough in cellular therapies show great promise (Fischbach et al., 2013) in the treatment of cardiovascular diseases, diabetes, corneal blindness and cystic fibrosis. Cellular therapy refers to the transfer of autologous or allogeneic cellular material into a patient for medical purposes. For example, in the regenerative setting stem cell-based therapies use somatic stem cells to repopulate damaged cells or reset tissue homeostasis. Somatic stem cells (SSCs, also known as adult stem cells) are a relatively rare cell population that resides in specialized cellular niches (Brunet et al., 2022). Our body uses SSCs as one way to repair tissues by replenishing lost or injured cells and by giving rise to progenitor or differentiated cells. Yet, we do not fully understand how these cells respond after an exposure to a stimulus, and which processes help to build biological (phenotypic) memories that impact human lifespan.

### SSCs and epigenetics

SSCs can undergo self-renewal processes and differentiate into specific cell types within their residential organs. To sustain body health, these SSCs play a crucial role in the maintenance of tissue homeostasis by replacing and replenishing exhausted cells as well as damaged cells resulted from environmental and pathological insults. For example, tissues with high turnover rates such as blood, skin, intestine, and bone marrow possess active SSCs (Post and Clevers, 2019). Conversely, tissues showing low turnover rates such as brain, stomach, and esophagus have small populations of stem cells (Sender and Milo, 2021). On the other hand, the liver and muscle will only regenerate upon injury (Tierney et al., 2018). However, over the course of one’s lifespan, stimuli, including lifestyle changes, exposure to infectious agents, injury, and aging (Ermolaeva et al., 2018), can also alter the SSCs niche and exhaust their regenerative potential, leading to a decline of life quality and increase of disease susceptibility. Consequently, stem cell aging and exhaustion contribute to the decline of cellular regenerative potential.

The cellular regenerative process is associated with the stem cell epigenome. This is because stem cell aging and exhaustion do not arise from changes in DNA sequences *per se* but rather emerge as altered memories in cellular programs (Ren et al., 2017; Ermolaeva et al., 2018). Besides, from a systems perspective the deterioration of cellular feedback-loop mechanisms during aging and tissue damage can diminish the cellular memory that maintains normal physiology (Kriete et al., 2010; Leung and Cotsarelis, 2022). This decline of regenerative health is also a whole-body trait that encompasses multi-organ memory, communication, and regulation. For instance, stem cell exhaustion is linked to diet (Hermetet et al., 2019), systemic milieu (blood environment) (Murphy and Thuret, 2015), microbiome (Tan et al., 2019), and even psychological stress can impact adult stem cell’s health (Amonoo et al., 2019). Thus, our ability to expand regenerative health to a systems level requires that we examine epigenetics and underlying mechanisms not just at the cellular level but also inspect how body-wide coordination can foster cellular and systems memories.

However, a key challenge is that the current definition of epigenetics is ambiguous (Greally, 2018) and lacks a formalism to describe how cellular and body memories are built. Currently, epigenetics focusses on chemical and structural modifications at DNA and chromatin levels. To surpass this challenge, we propose a new conceptual framework called Manifold Epigenetic Model (MEMo) that addresses how multi-organ interactions may confer memories to sustain a given whole-body trait, and how this principle can be utilized to enhance regenerative health.

Herein we focus on how SSCs and epigenetics contribute to cellular memories at the whole-body level. First, we will delve into evolving definitions of “epigenetics” and attempt to identify missing links. We will discuss how epigenetics and regenerative health are a whole-body phenomenon and share our perspectives on how we can utilize novel epigenetic concepts to devise our proposed Manifold Epigenetic Model (MEMo). Then we will enumerate engineering strategies that aim to boost tissue health, delay aging, and mitigate disease. Importantly, our new conceptual model can help shed new light on how to design interventions aiming to manipulate body-wide epigenetic mechanisms and restore regenerative health.

### The evolving definitions of epigenetics

**FIGURE 1:**
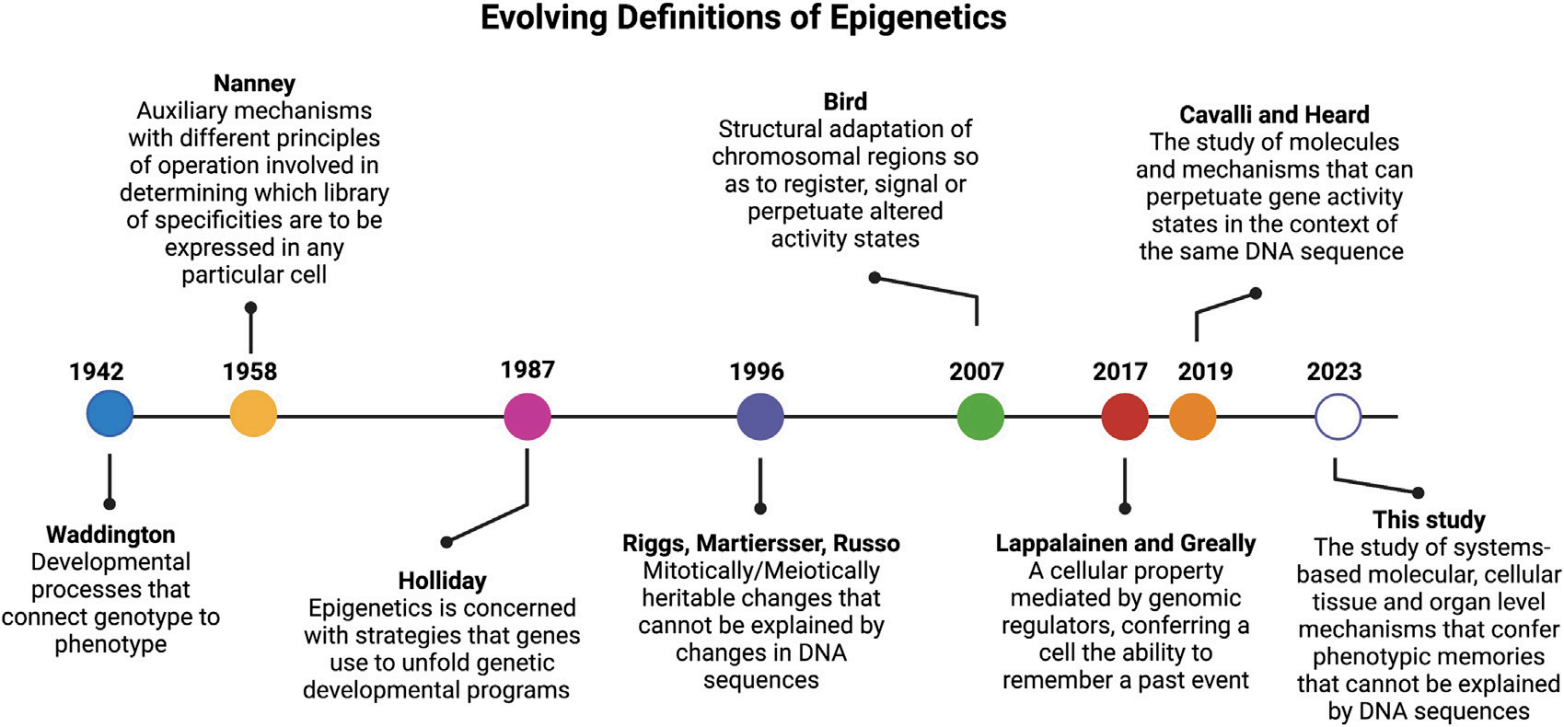
Timeline of epigenetic definitions since the term was first coined by Conrad Waddington in 1942. Updated epigenetics definitions reflect key discoveries and understanding over the past 80 years.

The landmark paper by Conrad Waddington in 1942 (Waddington, 1942a) (Figure 1) coined, for the first time, the term “epigenetics”, which was built on top of the concept of epigenesis (Lappalainen and Greally, 2017). In his original definition of epigenetics, Waddington attempted to merge embryology with genetics. Here the term “epigenetics” was defined as a “whole set of complex developmental processes that connect genotype and phenotype” (rephrased from the original paper). In this paper, Waddington further stated that one general feature of “epigenotype” is that “it consists of concatenations of processes linked together in a network”. Another insight from this classic paper, is that Waddington’s epigenetics was merely concerned with “complex developmental processes that connect genotype and phenotype” with no heritable traits. As rightly pointed out by Scott Gilbert (Gilbert, 2012), the prefix “epi” in this context is not associated with the Greek prefix for “above” or “beyond” as defined by most current studies. Actually, the prefix of “epi” coined by Waddington was used in the context of epigenesis, which referred to Aristotelian version of embryology theory where specialized tissue structures are developed from non-specialized precursors, in opposition to the preformationism that the development started with a miniature version of the organism in the gamete (Lappalainen and Greally, 2017). In short, the term “epigenetics” coined by Waddington is the marriage of embryology (epigenesis) and genetics.

More than a decade later, and to account for why cells with the same genetic materials may manifest different phenotypes, in 1958, David Nanney defined the term “epigenetic system” as “auxiliary mechanisms with different principles of operation involved in determining which [library] of specificities are to be expressed in any particular cells.” (Nanney, 1958) Nanney referred to the “library of specificities” as a template for replicating mechanisms based on DNA sequences. Thus, his definition hinted to the presence of cellular memory from past events.

In early 1980s (Feinberg and Vogelstein, 1983), discovery of the role of DNA methylation in cancer formation opened a new avenue to define epigenetics at the molecular level (Bird, 2002; Holliday, 2005). It is when the prefix “epi” of epigenetics acquired a new meaning from Greek root for “above” or “beyond” the genetics (i.e., the modifications are “above” or “on top of” DNA which is the genetic material). This definition of epigenetics was diverted from its original definition provided by Waddington that carried the context of epigenesis (i.e., developmental, and cellular memory).

Since then, the term epigenetics has become synonymous of DNA methylation (Bird, 2002; Holliday, 2005) and later was extended to include histone (Strahl and Allis, 2000; Jenuwein and Allis, 2001) and chromatin modifications (Margueron and Reinberg, 2010). In 1987, after more than a decade of his devotion to DNA methylation studies, Robin Holliday offered a more encompassing definition for epigenetics. He stated that “epigenetics is concerned with the strategy of the genes in unfolding the genetic program for development.” (Holliday, 1987) In 1996, Arthur Riggs and others defined epigenetics as “the study of mitotically and/or meiotically heritable changes in gene function that cannot be explained by changes in DNA sequences” (Russo et al., 1996). The elegance of this definition is that it tells what epigenetics is not and has since been passed on to later epigenetic definitions. For instance, Cavalli and Heard recently referred to epigenetics as “the study of molecules and mechanisms that can perpetuate alternative gene activity states in the context of the same DNA sequence.” (Cavalli and Heard, 2019)

However, the scope of epigenetics narrowed as it became more “molecularized” and recent definitions focused mostly on DNA and chromatin modifications. For instance, in 2007 Adrian Bird defined epigenetic events as “the structural adaptation of chromosomal regions so as to register, signal, or perpetuate altered activity states.” (Bird, 2007) In 2009, Berger and others provided an operational definition for epigenetics by referring an epigenetic trait as “a stably heritable phenotype resulting from changes in a chromosome without alterations in the DNA sequence” (Berger et al., 2009). In the past 2 decades, epigenetic studies have mainly focused on molecular mechanisms that altered DNA and chromosomal states (Allis and Jenuwein, 2016) and less emphasis was given to “memory” of cellular events. To remedy this limitation, Tuuli Lappalainen and John Greally recently defined epigenetic properties “as that of a cell, mediated by genomic regulators, conferring on the cell the ability to remember a past event.” (Lappalainen and Greally, 2017)

#### Epigenetics is more than just “above”, “on top of”, or “beyond” genetics

**FIGURE 2:**
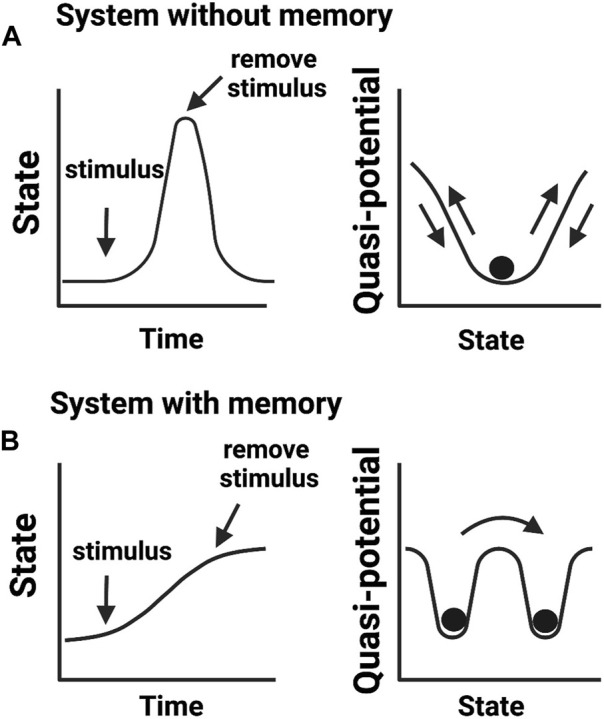
Biological memory in cellular systems. **(A)** Adaptive cellular response without a memory output. Upon encountering a stimulus (e.g., environmental stress), cells change their activities in response to altering conditions but return to a basal state in the absence of a stimulus. **(B)** Cellular response that confers memory. After encountering a stimulus, cells transit to a new state adapting to an imposed change. In this scenario, cells fail to return to the initial basal state even in the absence of an experienced stimulus. Cells transited to a new state but “remember” previous events. Right panels show quasi-potential energy landscapes (i.e., the energy barrier a cell needs to reorganize its intermolecular activities) associated with cellular memory states.

While these definitions of epigenetics consider dynamic changes in cellular states, they do not translate into knowledge of how cellular memories are formed. We argue that the concept of phenotypic memory (Figure 2) is vital for redefining epigenetics. This is because without memory, no phenotypic trait can be retained and therefore there will be no epigenetic mechanism to connect genotype to phenotype. This argument aligns well with the original vision provided by Waddington for “processes that connect genotype and phenotype”. Another concept described by Waddington was of “canalization” or the ability of a cell to select a canal (creode) at a bifurcation point and engage in cell-fate decision towards a developmental outcome (Waddington, 1942b). This idea of creodes permits a cell to decide its fate based on biological priors, for example, the presence of a mutation may set a cell to adopt a different fate. This notion has been recently expanded by Greally who stated that there are two types of epigenetic events: cellular reprogramming and different proportions of cell types slide through the variable use of “creodes” (polycreodism) (Greally, 2018). Our emphasis for phenotypic memory in the definition of epigenetics therefore agrees with the epigenetic events defined by Greally, who stresses that cellular memory is epigenetic.

However, a key difference from alternative views is that phenotypic memory is not necessarily a heritable trait, or mitotically/meiotically transmittable at cellular levels as pointed by Greally. For instance, the memory needed to sustain a phenotype acquired as an adaptive response to environmental stresses is not necessarily heritable (Ashapkin et al., 2020). Consequently, we opted not to include heritability as a mandatory criterion to define epigenetics, which opposes the most recent definitions of epigenetics (Bird, 2007; Deichmann, 2016). Hence, we reason that inclusion of heritability in defining epigenetics will be largely restrictive to the vigor of the field. We summarize “non-genetic mechanisms that confer diverse phenotypic memories under the same genotypic contexts” as a good statement that captures the essence of epigenetics.

In addition, phenotypic memory is not just initiated and maintained by “epigenetic” modifications such as DNA methylation and histone modifications at the single gene level, or other mechanisms that have been recently aggregated under the same umbrella of epigenetics, such as the inclusion of RNA-based gene regulation (Matzke and Mosher, 2014; Holoch and Moazed, 2015) and three-dimensional chromosomal architecture (Zheng and Xie, 2019). Indeed, phenotypic memory is an emergent property, namely, a property arising from the collective action of genes. Actually, the concept of epigenetics originally proposed by Waddington carried a dynamic network view of development (Waddington, 1942a; Jablonka and Lamm, 2012). He stated that one general feature of epigenotype is that “it consists of concatenations of processes linked together in a network”. This insightful foresight has been proved seminal with the advances of systems biology around 2000 (Kitano, 2002). With insights learned from systems biology, Angela Pisco, Aymeric d’Hérouël, and Sui Huang have elegantly elaborated that the “epigenetic landscape” in cell fate determination is more than a metaphor and has specific mathematical foundations and the “epigenetic memory” that maintain a cell type identity has its root in gene regulatory network in producing multiple stable states (also called “attractor” states) (Pisco et al., 2016). Furthermore, self-sustained feedback loops in biological networks are crucial elements for a biological system, such as a cell to “remember” a past event, even when the signal that initiates the memory has been withdrawn for a certain period or is absent (Ptashne, 2007).

A main conclusion drawn from the above arguments is that coupling of different epigenetic layers goes beyond DNA modification and includes distinct transcriptional gene regulation and feedback mechanisms to bridge genotype to phenotype. Thus, this framework discriminates hierarchical levels of regulation, a mechanism often used when a biological system prioritizes multiple objectives with the help of tunable functions. Another of such examples comes from the olfactory system. In fact, this is a conundrum in neuroscience, how monoallelic and diverse expression of olfactory receptors (OR) can be sustained and at the same time safeguard improper stimulation and wiring of the olfactory system that would lead to misinterpretation of chemical signals (Magklara and Lomvardas, 2013). To address this question, Tian *et al* utilized information on OR expression, and constructed a comprehensive mathematical model that captures all physical interaction components of OR transcription. Their computational modeling study shows that a coordinated and evolutionarily optimized three-layered regulation mechanism, controls the expression of each OR gene by coupling histone modifications, enhancer competition, and negative feedback (Tian et al., 2016). As such, multilayer coupling can efficiently modulate genes with similar biological roles and simultaneously maintain circuit homeostasis.

### Expanding epigenetics

To circumvent the above-mentioned limitations, we provide an operational definition of epigenetics by adhering to Waddington’s definition and yet capture contemporary understanding of molecular genetics and systems biology, especially the property of biological systems such as cells to “remember” past events (Bonasio et al., 2010). Since heritability is not a mandatory criterion to define epigenetics, we redefine epigenetics as “the study of systems-based molecular and cellular mechanisms that confer phenotypic memories that cannot be explained by changes in DNA sequences”.

By including systems-based mechanisms in this definition we highlight the role of networks and feedback-loop mechanisms in coupling several epigenetic layers and forge phenotypic memories. Also, the inclusion of both molecular and cellular mechanisms that encompass chromatin modifications, biomolecular interactions, and cell-cell communications serve as bridges to formulate a new conceptual model on body-wide phenotypic memories as described below.

#### Aging is a multi-organ process and youthfulness is a state maintained by body-wide memory

Numerous links between aging and epigenetics are known. Yet, current epigenetic models fail to account how body-wide memory maintains a youthful state. Aging is an unavoidable and gradually decline of functional and homeostatic integrity over the lifespan of an individual. Aging is not an isolated biological event but a whole-body process that impacts multi-organ communication and homeostasis. Currently, well acknowledged hallmarks of aging are mainly genetic and cellular (Lopez-Otin et al., 2013). These include genomic instability, epigenetic alterations, telomere attrition, stem cell exhaustion, cellular senescence, mitochondrial dysfunction, deregulated nutrient sensing, loss of proteostasis, and altered intercellular communication (Lopez-Otin et al., 2013).

However, multi-organ failure has been seen as a clinical symptom in elderly patients (Wang and Fan, 1990). Further, a recent study using high-resolution isotope microscopy imaging to measure the age of cells and proteins found that adult mouse organs are composed of mosaics of cells that span different ages (Arrojo et al., 2019). The finding of age mosaicism across multiple organs is unexpected. This is because tissues, such as skin, that is constantly being replaced throughout a lifetime, are expected to be “young”. Whereas tissues, such as the brain, that has minimal or no turnover, is expected to reflect an individual’s age and long-lived proteins in these low-turnover tissues are expected to decline with aging. Surprisingly, this study showed that most organs consist of mixed cells and proteins of vastly different ages regardless of their regeneration potential. This observation strongly suggests that aging involves inter-organ communication and the regeneration of tissues in one organ requires cues released from another organ. As such, we reason that “altered inter-organ communication” is a hallmark of aging that is currently missing in our list. Overall, a better understanding of epigenetic mechanisms that mediate body-wide memory is needed to augment our ability of enhancing cellular resilience, alleviating aging, and identifying strategies that can sustain homoeostatic responses to expand healthspan.

Although aging is a progressive process over a lifespan, there are epigenetic memory mechanisms that can maintain and resist pathological aging, such as in progeria (Burtner and Kennedy, 2010). The most persuasive evidence comes from heterochronic parabiosis studies, in which investigators exposed old mice to a young systemic (i.e., blood) environment (Conboy et al., 2005), and showed that youthfulness is a body-wide phenotypic state involving memory mechanisms throughout the body. The presence of young systemic factors in the blood helped to rejuvenate vascular (Katsimpardi et al., 2014), neuron regenerative and cognitive functions in aged mice (Villeda et al., 2014), while factors from old blood negatively regulated cognitive function (Villeda et al., 2011) and impacted multiple tissues (Rebo et al., 2016). These studies suggest a key role of “young” systemic factors in the renewal of body-wide memories and restoration of a youthful state, while factors from old blood counteract by disrupting these memories.

However, geroscience, the study of aging, is mainly focused on genetic and molecular bases (Fontana et al., 2010; Singh et al., 2019). Even the hallmarks of aging (Lopez-Otin et al., 2013) lack aspects of whole body epigenetic regulation as described above. Hence, there is a need to better understand why we age and how epigenetic memory of youth is established at the body-wide level to effectively restore healthy and young state and slow down aging process.

##### Manifold epigenetics: A conceptual model to convey for body-wide phenotypic memories

**FIGURE 3:**
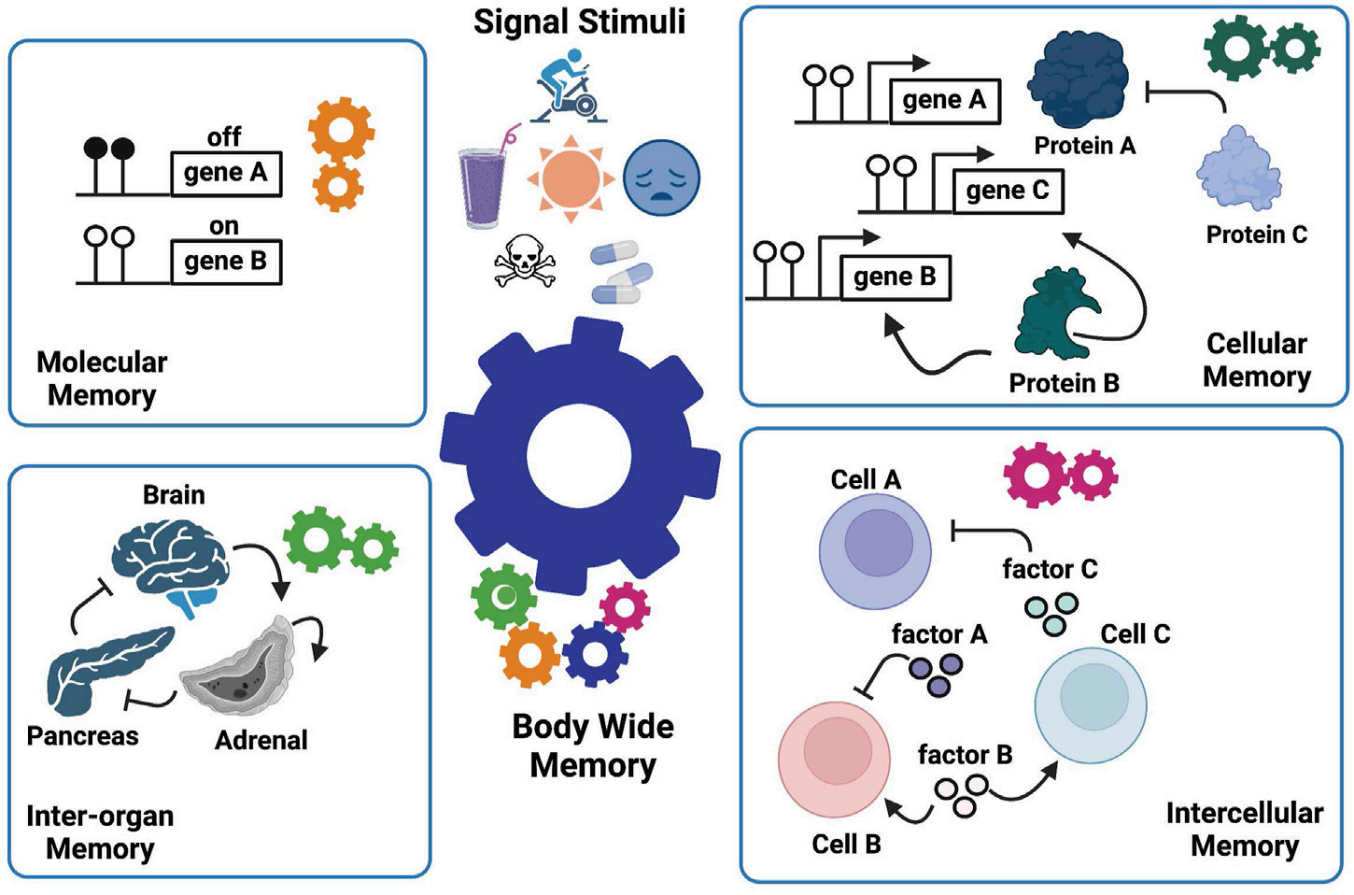
The Manifold Epigenetic Model—MEMo. This model proposes that epigenetic biologic memory is an emergent property arising from a variety of systems-based interactions between different components across several biological levels (cells, tissues, and organs). The whole-body phenotypic memory (e.g., youthfulness) is the response to external (drugs, toxicants, environment, diet, physical activity, and mental stress) and developmental cues, and represents the orchestrated output from molecular, cellular, intercellular, and inter-organ memories. As illustrated, the epigenetic memory relies on positive feedback loops in each biological layer to enforce persistent signals that sustain a given biological state.

As illustrated above, the current epigenetic models which focus mainly on DNA and chromatin modifications are unable to explain how whole-body memories arise. Thus, we extended our definition of epigenetics to formulate a conceptual model called Manifold Epigenetic Model (MEMo). Here, manifold epigenetics is defined as “the study that concerns with the totality of molecular, cellular, and environmental systems-based mechanisms that confer body-wide phenotypic memories without altering DNA sequences”. In other words, any outputs of non-genetic systems that confer body-wide phenotypic memories are within the mechanistic modes of manifold epigenetics. We borrow the term “manifold” from mathematics that deals with multidimensional topology, to refer to multifaceted and multilayered nature of epigenetic mechanisms that regulate body-wide phenotypic memories (Figure 3). MEMo also states that a whole body phenotypic memory is an emergent property arising from the collective coordination of epigenetic mechanisms derived from multiple layers of systems activities. At the heart of these memory mechanisms is the presence of positive feedback and/or feedforward loops that sustain and stabilize a given phenotypic state throughout biological systems (Xiong and Ferrell, 2003; Brandman et al., 2005; Natoli and Ostuni, 2019; Takamiya et al., 2021) even in the absence of stimulating cues.

MEMo further indicates that the entity, be it a molecule, a cell, or an organ that mediates the positive feedback, is the effective component enabling a biological system to “remember” (i.e., sustain) a phenotypic trait. We called these key components “epigenetic memory enforcers”. For instance, gene B, cell B, and adrenal organ illustrated in Figure 3 are memory enforcers. In other words, MEMo predicts that the epigenetic state of a cell or an organ is modulated by the epigenetic state of corresponding memory enforcers in the network form. Furthermore, the epigenetic state of a memory enforcer at a given biological level (e.g., organ level) is also dependent on the epigenetic state of a memory enforcer at another biological level (e.g., cell level). Such multileveled dependency of epigenetic memory states forms the basis of the “manifoldness” of epigenetic mechanisms to confer body-wide phenotypic memories. The essence of MEMo is to view health and disease under the light of networks to build a body-wide memory.

## Manifold Epigenetic Engineering: A guided principle to exploit manifold epigenetic mechanisms

MEMo provides a conceptual framework to formulate a guided principle called - Manifold Epigenetic Engineering (MEE) that allow us to manipulate epigenetic memories underlying whole-body context. Here, we use the term “engineering” to mean “manipulation design”. MEE is defined by “non-genetic design manipulations that exploit manifold epigenetic mechanisms to dismantle noxious phenotypic memories while re-establishing beneficial body-wide phenotypic memories”. Although still far from being practical at the current stage, MEE nonetheless provides a feasible guiding principle to design novel treatment strategies based on manifold perspectives in medicine (Ung et al., 2022).

Under the MEMo framework, memory enforcers are key targets for biological manipulation. For example, in a pathological state, memory enforcers arising but not solely from exhausted cells, which play key roles in shaping intercellular communication processes can drive epigenetic memory. By stabilizing certain phenotypic states through positive feedback loops, exhausted cells can support epigenetic memory in disease, and they themselves are targets for engineering manipulations aiming to restore healthy homeostasis.

The first step in MEE is therefore to identify memory enforcers that are crucial for a given phenotypic memory. A hierarchical approach can be used to decipher key players. We reason those interactions among organs throughout the body exert more global impact on epigenetic memory than the cell-cell communication in local cellular niche. The same reason could also hold true for the intercellular communication in a cellular niche which has broader impact on epigenetic memory than intermolecular interactions conferring epigenetic memory in any cell in that niche. As such, we propose an “embedding” strategy to rank the importance of memory enforcers. First, the organ(s) that act as memory enforcer for a body-wide phenotype are deemed the target organ. Next, cell types that act as memory enforcers within the target organ are deemed as target cells. It is these target cells the main intervention subjects for MEE because they are the key players that establish epigenetic memories at the target organs that in turn sustain body-wide epigenetic memories.

### Reviving body-wide regenerative health *via* MEE

**FIGURE 4:**
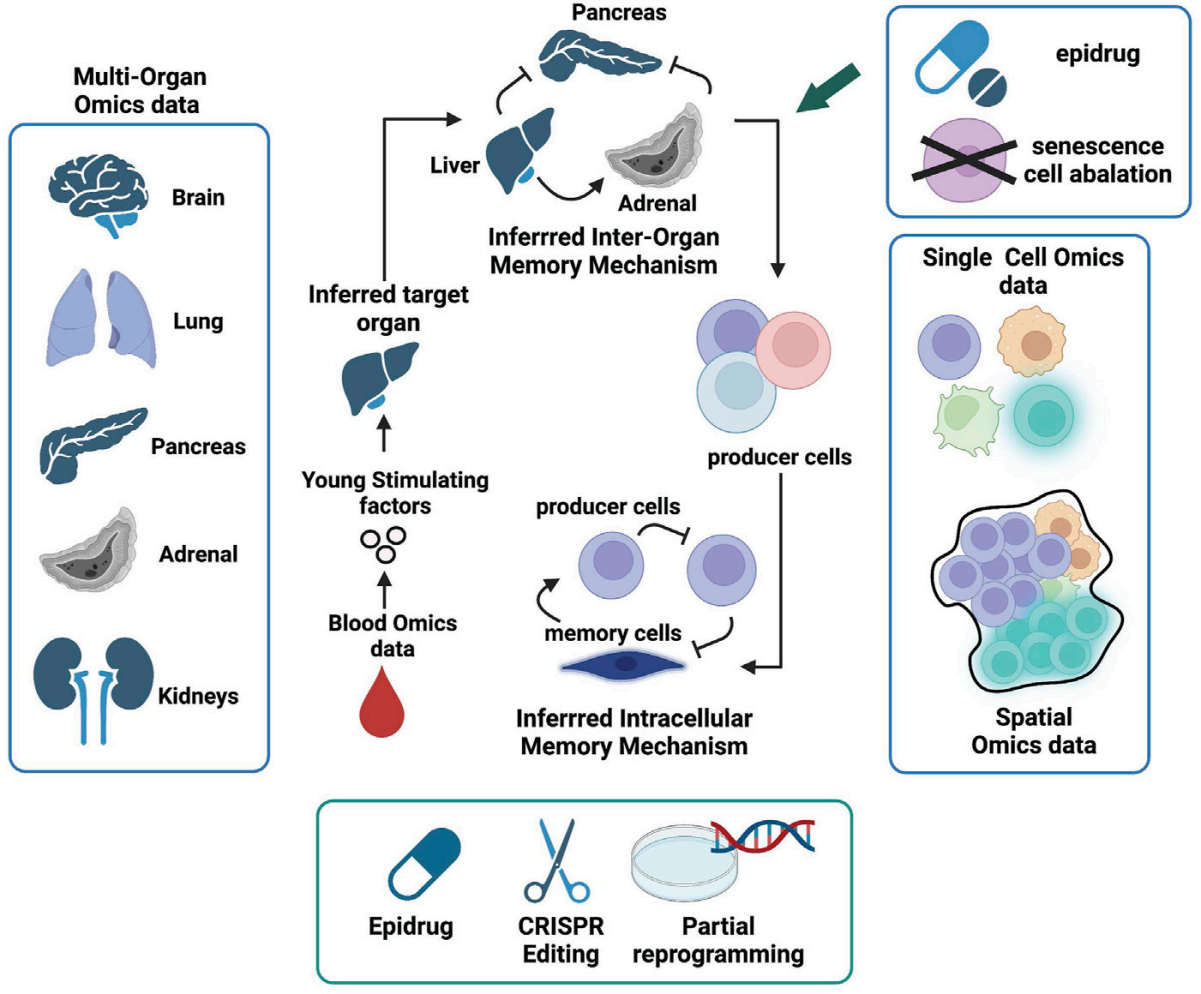
Manifold Epigenetic Engineering (MEE). Schematic representation of key omic datasets and strategies to achieve MEE and invigorate whole body regenerative health. The first MEE step is to identify circulating systemic factors present in higher levels in young but decreased or absent in aged individuals. Next, multi-organ single-cell omics and spatial data can be used to identify cells that produce “young” factors and infer their communication networks within cellular niches using knowledge from receptor-ligand interactions. Organs that produce “young” factors and are also involved in positive feedback interactions are deemed memory enforcers and will be prioritized as target organs. Cell types that mediate positive feedbacks in the communication webs are deemed memory enforcers and will be prioritized as target cells. Both target organs and cells will be subjected to engineered interventions (senescence cell ablation, drug intervention, chromatin state editing, and partial reprogramming) in aged individuals to help restore regenerative health.

To restore regenerative health *via* MEE (Figure 4), we first need to identify organ-wise epigenetic memory enforcers that produce molecular factors that sustain activities, such as tissue repair and stem cell regeneration at the inter-organ level. For example, the clues for the “fountain of youth”, as indicated by heterochronic parabiosis studies, may be present in young blood. Proteomics and metabolomics data derived from young and old blood (Emilsson et al., 2018; Bar et al., 2020) are valuable resources to find such factors.

We also advocate for blood-borne factors being part of the internal *milieu* (environment) that encodes body-wide functional states regardless of local intercellular signaling events and homeostatic states. Deciphering such blood-borne young-stimulating factors is a promising and approachable strategy to identify body-wide epigenetic regulating factors and move towards a better regenerative health. A starting point to sort out blood-borne young-stimulating factors from other irrelevant factors is to identify secreted factors (peptides, proteins, RNAs, etc*.*) whose expression levels are not just elevated in young blood, but presumably low or even absent in old blood. The next criterion is to inspect whether the level of any of these blood-borne young-stimulating factors correlates with the expression of receptors such as adenosine receptors (Feoktistov et al., 2009), nerve growth factor receptor (NGFR) (Liu et al., 2021), and apelin receptors (Kang and Yang, 2020) whose downstream activities have been associated with youth-related pathways such as tissue repair and remodeling (Ninov and Yun, 2015), and regulation of mitochondrial metabolism (Singh, 2021). Here, systems-based approaches such as Quantitative Endocrine Network Interaction Estimation (QENIE) developed by Seldin et al. (2018) can be employed to infer such ligand-receptor correlations at the whole-body level.

The next task is to find organs or tissues that produce these “young-stimulating” factors. This requires the availability of multi-organ omics data across different aging stages for both healthy and disease states. Although such data are rare and currently it is very challenging to obtain the multi-organ omics data from human (Tabula Sapiens et al., 2022), there are increasing multi-organ omics data generated from mouse models over past few years (Kozawa et al., 2020; Schaum et al., 2020; Rumienczyk et al., 2021). Organs and/or tissues that make circulating “young-stimulating” factors could be identified by comparing multi-organ gene expression profiles at different age groups. The inter-organ crosstalk with respect to young and old stages can be inferred from expression of receptors and their ligands across different organs (Rana et al., 2012). The “young” blood factors derived from organs/tissues that are involved in positive feedback loops in the inter-organ crosstalk are deemed epigenetic memory enforcers for “youthful” phenotype and will become the target organs/tissues for reviving regenerative health *via* MEE.

The next stage is to find cellular niches inhabiting target organs/tissues that act as epigenetic memory enforcers. Thanks to the advancement of single-cell and spatial transcriptomics technologies (Stuart and Satija, 2019; Moffitt et al., 2022) and the Cell Atlas initiative (Elmentaite et al., 2022), we can now integrate these two layers of omics data to reveal intercellular dynamics within cellular niches across different organs (Longo et al., 2021) and decipher cell-cell communications that build epigenetic memories (Armingol et al., 2021). From the receptor-ligand expression profiles (Efremova et al., 2020), we can dissect intercellular communications and identify target cells, i.e., cell types that constitute positive feedback loops in the intercellular memory to make young-stimulating factors, maintaining the youthful phenotype of target organs. These target cells will be the manipulating subjects of MEE. We hypothesize that these intercellular communication web consists of both producers of young-stimulating factor and different types of stem cells, including SSCs.

After identifying target cells, the subsequent task is to prioritize manipulation approaches to engineer the desired epigenetic states in these cells. The goal is to reactivate the function of target cells in older individuals. Restored target cells and organs will then replenish blood-borne young-stimulating factors to re-establish homeostasis and body-wide epigenetic memories that collectively sustain youthful phenotypes equipped with healthy regenerative capabilities. Toward the goal of replenishing regenerative health, both general and engineered strategies are needed. Below we briefly outline these two intervention types.

### General interventions

General interventions promote conducive biological environments, such as nurturing cellular niches, that sustain the rehabilitation of stem cells, and repair of injured or aging tissues towards a normal state. Although non-specific, general interventions can nonetheless facilitate the efficacy of engineered interventions to promote regenerative health. General interventions include exercise, healthy diets, sleep, and a healthy mental state. The health benefits of exercise are well recognized (Neufer et al., 2015) and physical exercise is a mean to protect mitochondrial health (Sorriento et al., 2021), since mitochondrial dysfunction is associated with aging (Amorim et al., 2022). Fasting-mimicking diets (low in calories, sugars, and protein but high in unsaturated fats) have been shown to promote multisystem regeneration and improve cognitive performance (Brandhorst et al., 2015) as well as reducing the risk of diabetes, cancer, and cardiovascular disease (Wei et al., 2017). Sleep drives the clearance of metabolites and protein aggregates in the brain and helps prevent neurological disorders (Xie et al., 2013; Tarasoff-Conway et al., 2015), such as Alzheimer’s disease, an age-related disease. Good mental health is important to maintain healthy brain-body pathways to sustain body-wide homeostasis (Gianaros and Wager, 2015; Hoogendoorn et al., 2017). Hence, general interventions provide a general, non-specific body-wide setting to facilitate the outcome of engineered interventions to promote the restoration of regenerative health.

### Engineered interventions

It is the target organ and target cells the subjects of engineered interventions. Indeed, manipulation of activities of these target organs/cells will augment epigenetic memories towards a given body-wide phenotype. Since target organs are those involved in establishing body-wide epigenetic memories, repairing their dysregulated states can help restore tissue regenerative health. In principle, this can be achieved by ablating senescent cells in these organs *via* senolytic drugs (Robbins et al., 2021) or through engineered senolytic chimeric antigen receptor (CAR) T-cells (Amor et al., 2020). Engineered interventions on target cells include drug-based targeting, chromatin state editing, and *ex vivo* partial reprogramming. The epigenetic memory states for these target cells can be manipulated *via* epigenetic drugs (or epidrugs) (Berdasco and Esteller, 2019) that modify DNA methylation or histone modification states in the cells. Furthermore, drugs that alter the activities of target proteins, such as those acting as memory enforcers in maintaining cellular memories, can be employed. Beside drugs, chromatin states of target cells can also be manipulated *via* CRISPR-based genome editing approaches (Nakamura et al., 2021).

Recent studies showed that partial reprogramming by ectopic expression of Yamanaka factors can ameliorate age-associated epigenetic hallmarks (Ocampo et al., 2016) and recover youthful epigenetic states (Lu et al., 2020). The Yamanaka factors consist of four transcription factors OCT4, SOX2, KLF4, and MYC (usually simplified as OSKM) (Takahashi and Yamanaka, 2006). Technically, it is feasible to conduct ectopic expression of OSKM *via* engineered genetic constructs introduced into organoid culture of aged tissues with cellular niches consist of target cells, including somatic stem cells derived from patients. The reprogrammed tissues will then be transplanted back to the donors. The “rejuvenated” memory enforcing cells with reprogrammed epigenetic states will release signaling factors to alter cell-cell communications and ultimately inter-organ crosstalk, leading to the restoration of a healthy regenerative state.

## Conclusion

We have briefly reviewed the historical context of epigenetics, identified gaps in current definitions and provided a rationale to include phenotypic memory in the context of epigenetics. We expand this concept to the whole body and recognize the need for a new conceptual framework to address body-wide epigenetic memory. MEMo, as such a novel conceptual framework, could address how epigenetic memory mechanisms emerge and encompass cellular, tissues, and feedback loops to generate system (body-wide) phenotypes. In addition, MEMo provides a practical platform—such as MEE, which is capable of integrating engineered manipulations to rehabilitate organismal traits, including youthfulness (or regenerative health). These principles better reflect disease etiology by moving from a gene-centric to a systems-level view and considering the emergent properties that arise from the interplay of multiple types of cells and organs throughout the body.

We reason that phenotypic memory underpins epigenetic mechanisms and holds promise for a new chapter in biomedical research. To our knowledge, there is no conceptual model that provides mechanistic insights to explain how body-wide phenotypic memories are built, albeit the existence of multiscale models that incorporate events across different biological levels (Wolkenhauer et al., 2014). Hence, the redefined epigenetics and formulation of MEMo opens a new avenue to understand disease pathogenesis and provides guidance for innovative treatment. In particular, reviving body-wide regenerative health *via* MEE will be a beneficial remedy for broad types of diseases. Our current focus is on regenerative health, but nonetheless MEMo is generalizable to many other biological conditions and cell types. This is because epigenetic memories are ubiquitously present to sustain stability and functionality of both physiological and pathological states. In particular, T-cells that play pivotal roles in adaptive immunity, are in many aspects similar to stem cells (Bhandoola and Sambandam, 2006) such as stemness, lineage commitment, and cell exhaustion. As such, MEE can also be applied to engineer immunity in elderly patients and reboot their immune response.

Although, we are still far from realizing the full vision of MEE, our concept nonetheless provides a roadmap for future works, in particular the awareness of the importance of multi-organ omic data across the lifespan in disease and healthy animal models, and the need to develop integrative engineering approaches for better manipulation of whole-body phenotypes, such as aging.

## Data Availability

The original contributions presented in the study are included in the article/supplementary material, further inquiries can be directed to the corresponding authors.

## References

[B1] Allis C. D. Jenuwein T. (2016). The molecular hallmarks of epigenetic control. Nat. Rev. Genet. 17, 487–500. 10.1038/nrg.2016.59 27346641

[B2] Amonoo H. L. Massey C. N. Freedman M. E. El-Jawahri A. Vitagliano H. L. Pirl W. F. (2019). Psychological considerations in hematopoietic stem cell transplantation. Psychosomatics 60, 331–342. 10.1016/j.psym.2019.02.004 31072626 PMC6626677

[B3] Amor C. Feucht J. Leibold J. Ho Y. J. Zhu C. Alonso-Curbelo D. (2020). Senolytic CAR T cells reverse senescence-associated pathologies. Nature 583, 127–132. 10.1038/s41586-020-2403-9 32555459 PMC7583560

[B4] Amorim J. A. Coppotelli G. Rolo A. P. Palmeira C. M. Ross J. M. Sinclair D. A. (2022). Mitochondrial and metabolic dysfunction in ageing and age-related diseases. Nat. Rev. Endocrinol. 18, 243–258. 10.1038/s41574-021-00626-7 35145250 PMC9059418

[B5] Armingol E. Officer A. Harismendy O. Lewis N. E. (2021). Deciphering cell-cell interactions and communication from gene expression. Nat. Rev. Genet. 22, 71–88. 10.1038/s41576-020-00292-x 33168968 PMC7649713

[B6] Arrojo E. D. R. Lev-Ram V. Tyagi S. Ramachandra R. Deerinck T. Bushong E. (2019). Age mosaicism across multiple scales in adult tissues. Cell Metab. 30, 343–351. 10.1016/j.cmet.2019.05.010 31178361 PMC7289515

[B7] Ashapkin V. V. Kutueva L. I. Aleksandrushkina N. I. Vanyushin B. F. (2020). Epigenetic mechanisms of plant adaptation to biotic and abiotic stresses. Int. J. Mol. Sci. 21, 7457. 10.3390/ijms21207457 33050358 PMC7589735

[B8] Bar N. Korem T. Weissbrod O. Zeevi D. Rothschild D. Leviatan S. (2020). A reference map of potential determinants for the human serum metabolome. Nature 588, 135–140. 10.1038/s41586-020-2896-2 33177712

[B9] Berdasco M. Esteller M. (2019). Clinical epigenetics: Seizing opportunities for translation. Nat. Rev. Genet. 20, 109–127. 10.1038/s41576-018-0074-2 30479381

[B10] Berger S. L. Kouzarides T. Shiekhattar R. Shilatifard A. (2009). An operational definition of epigenetics. Genes Dev. 23, 781–783. 10.1101/gad.1787609 19339683 PMC3959995

[B11] Bhandoola A. Sambandam A. (2006). From stem cell to T cell: One route or many? Nat. Rev. Immunol. 6, 117–126. 10.1038/nri1778 16491136

[B12] Bird A. (2002). DNA methylation patterns and epigenetic memory. Genes Dev. 16, 6–21. 10.1101/gad.947102 11782440

[B13] Bird A. (2007). Perceptions of epigenetics. Nature 447, 396–398. 10.1038/nature05913 17522671

[B14] Bonasio R. Tu S. Reinberg D. (2010). Molecular signals of epigenetic states. Science 330, 612–616. 10.1126/science.1191078 21030644 PMC3772643

[B15] Brandhorst S. Choi I. Y. Wei M. Cheng C. W. Sedrakyan S. Navarrete G. (2015). A periodic diet that mimics fasting promotes multi-system regeneration, enhanced cognitive performance, and healthspan. Cell Metab. 22, 86–99. 10.1016/j.cmet.2015.05.012 26094889 PMC4509734

[B16] Brandman O. Ferrell J. E. Jr. Li R. Meyer T. (2005). Interlinked fast and slow positive feedback loops drive reliable cell decisions. Science 310, 496–498. 10.1126/science.1113834 16239477 PMC3175767

[B17] Brunet A. Goodell M. A. Rando T. A. (2022). Ageing and rejuvenation of tissue stem cells and their niches. Nat. Rev. Mol. Cell Biol. 24, 45–62. 10.1038/s41580-022-00510-w 35859206 PMC9879573

[B18] Burtner C. R. Kennedy B. K. (2010). Progeria syndromes and ageing: What is the connection? Nat. Rev. Mol. Cell Biol. 11, 567–578. 10.1038/nrm2944 20651707

[B19] Cavalli G. Heard E. (2019). Advances in epigenetics link genetics to the environment and disease. Nature 571, 489–499. 10.1038/s41586-019-1411-0 31341302

[B20] Conboy I. M. Conboy M. J. Wagers A. J. Girma E. R. Weissman I. L. Rando T. A. (2005). Rejuvenation of aged progenitor cells by exposure to a young systemic environment. Nature 433, 760–764. 10.1038/nature03260 15716955

[B21] Culme-Seymour E. J. Davie N. L. Brindley D. A. Edwards-Parton S. Mason C. (2012). A decade of cell therapy clinical trials (2000-2010). Regen. Med. 7, 455–462. 10.2217/rme.12.45 22817619

[B22] Deichmann U. (2016). Epigenetics: The origins and evolution of a fashionable topic. Dev. Biol. 416, 249–254. 10.1016/j.ydbio.2016.06.005 27291929

[B23] Efremova M. Vento-Tormo M. Teichmann S. A. Vento-Tormo R. (2020). CellPhoneDB: Inferring cell-cell communication from combined expression of multi-subunit ligand-receptor complexes. Nat. Protoc. 15, 1484–1506. 10.1038/s41596-020-0292-x 32103204

[B24] Elmentaite R. Dominguez Conde C. Yang L. Teichmann S. A. (2022). Single-cell atlases: Shared and tissue-specific cell types across human organs. Nat. Rev. Genet. 23, 395–410. 10.1038/s41576-022-00449-w 35217821

[B25] Emilsson V. Ilkov M. Lamb J. R. Finkel N. Gudmundsson E. F. Pitts R. (2018). Co-regulatory networks of human serum proteins link genetics to disease. Science 361, 769–773. 10.1126/science.aaq1327 30072576 PMC6190714

[B26] Ermolaeva M. Neri F. Ori A. Rudolph K. L. (2018). Cellular and epigenetic drivers of stem cell ageing. Nat. Rev. Mol. Cell Biol. 19, 594–610. 10.1038/s41580-018-0020-3 29858605

[B27] Feinberg A. P. Vogelstein B. (1983). Hypomethylation distinguishes genes of some human cancers from their normal counterparts. Nature 301, 89–92. 10.1038/301089a0 6185846

[B28] Feoktistov I. Biaggioni I. Cronstein B. N. (2009). Adenosine receptors in wound healing, fibrosis and angiogenesis. Handb. Exp. Pharmacol. 193, 383–397. 10.1007/978-3-540-89615-9_13 PMC3729032 19639289

[B29] Fischbach M. A. Bluestone J. A. Lim W. A. (2013). Cell-based therapeutics: The next pillar of medicine. Sci. Transl. Med. 5, 179ps7. 10.1126/scitranslmed.3005568 PMC3772767 23552369

[B30] Fontana L. Partridge L. Longo V. D. (2010). Extending healthy life span--from yeast to humans. Science 328, 321–326. 10.1126/science.1172539 20395504 PMC3607354

[B31] Gianaros P. J. Wager T. D. (2015). Brain-body pathways linking psychological stress and physical health. Curr. Dir. Psychol. Sci. 24, 313–321. 10.1177/0963721415581476 26279608 PMC4535428

[B32] Gilbert C. Tang T. C. Ott W. Dorr B. A. Shaw W. M. Sun G. L. (2021). Living materials with programmable functionalities grown from engineered microbial co-cultures. Nat. Mater 20, 691–700. 10.1038/s41563-020-00857-5 33432140

[B33] Gilbert S. F. (2012). Commentary: 'The epigenotype' by C.H. Waddington. Int. J. Epidemiol. 41, 20–23. 10.1093/ije/dyr186 22253306

[B34] Greally J. M. (2018). A user's guide to the ambiguous word 'epigenetics. Nat. Rev. Mol. Cell Biol. 19, 207–208. 10.1038/nrm.2017.135 29339796

[B35] Hermetet F. Buffiere A. Aznague A. Pais de Barros J. P. Bastie J. N. Delva L. (2019). High-fat diet disturbs lipid raft/TGF-beta signaling-mediated maintenance of hematopoietic stem cells in mouse bone marrow. Nat. Commun. 10, 523. 10.1038/s41467-018-08228-0 30705272 PMC6355776

[B36] Hofer M. Lutolf M. P. (2021). Engineering organoids. Nat. Rev. Mater 6, 402–420. 10.1038/s41578-021-00279-y 33623712 PMC7893133

[B37] Holliday R. (2005). DNA methylation and epigenotypes. Biochem. (Mosc) 70, 500–504. 10.1007/s10541-005-0144-x 15948704

[B38] Holliday R. (1987). The inheritance of epigenetic defects. Science 238, 163–170. 10.1126/science.3310230 3310230

[B39] Holoch D. Moazed D. (2015). RNA-mediated epigenetic regulation of gene expression. Nat. Rev. Genet. 16, 71–84. 10.1038/nrg3863 25554358 PMC4376354

[B40] Hoogendoorn C. J. Roy J. F. Gonzalez J. S. (2017). Shared dysregulation of homeostatic brain-body pathways in depression and type 2 diabetes. Curr. Diab Rep. 17, 90. 10.1007/s11892-017-0923-y 28815394 PMC5993206

[B41] Jablonka E. Lamm E. (2012). Commentary: The epigenotype--a dynamic network view of development. Int. J. Epidemiol. 41, 16–20. 10.1093/ije/dyr185 22253305

[B42] Jenuwein T. Allis C. D. (2001). Translating the histone code. Science 293, 1074–1080. 10.1126/science.1063127 11498575

[B43] Kang J. S. Yang Y. R. (2020). Circulating plasma factors involved in rejuvenation. Aging (Albany NY) 12, 23394–23408. 10.18632/aging.103933 33197235 PMC7746393

[B44] Katsimpardi L. Litterman N. K. Schein P. A. Miller C. M. Loffredo F. S. Wojtkiewicz G. R. (2014). Vascular and neurogenic rejuvenation of the aging mouse brain by young systemic factors. Science 344, 630–634. 10.1126/science.1251141 24797482 PMC4123747

[B45] Kim J. Campbell A. S. de Avila B. E. Wang J. (2019). Wearable biosensors for healthcare monitoring. Nat. Biotechnol. 37, 389–406. 10.1038/s41587-019-0045-y 30804534 PMC8183422

[B46] Kitano H. (2002). Systems biology: A brief overview. Science 295, 1662–1664. 10.1126/science.1069492 11872829

[B47] Kozawa S. Sagawa F. Endo S. De Almeida G. M. Mitsuishi Y. Sato T. N. (2020). Predicting human clinical outcomes using mouse multi-organ transcriptome. iScience 23, 100791. 10.1016/j.isci.2019.100791 31928967 PMC7033637

[B48] Kriete A. Bosl W. J. Booker G. (2010). Rule-based cell systems model of aging using feedback loop motifs mediated by stress responses. PLoS Comput. Biol. 6, e1000820. 10.1371/journal.pcbi.1000820 20585546 PMC2887462

[B49] Lappalainen T. Greally J. M. (2017). Associating cellular epigenetic models with human phenotypes. Nat. Rev. Genet. 18, 441–451. 10.1038/nrg.2017.32 28555657

[B50] Leung T. H. Cotsarelis G. (2022). Cellular memories - more than skin deep. N. Engl. J. Med. 386, 793–795. 10.1056/NEJMcibr2118516 35196433

[B51] Liu Z. Wu H. Huang S. (2021). Role of NGF and its receptors in wound healing (Review). Exp. Ther. Med. 21, 599. 10.3892/etm.2021.10031 33884037 PMC8056114

[B52] Longo S. K. Guo M. G. Ji A. L. Khavari P. A. (2021). Integrating single-cell and spatial transcriptomics to elucidate intercellular tissue dynamics. Nat. Rev. Genet. 22, 627–644. 10.1038/s41576-021-00370-8 34145435 PMC9888017

[B53] Lopez-Otin C. Blasco M. A. Partridge L. Serrano M. Kroemer G. (2013). The hallmarks of aging. Cell 153, 1194–1217. 10.1016/j.cell.2013.05.039 23746838 PMC3836174

[B54] Lu Y. Brommer B. Tian X. Krishnan A. Meer M. Wang C. (2020). Reprogramming to recover youthful epigenetic information and restore vision. Nature 588, 124–129. 10.1038/s41586-020-2975-4 33268865 PMC7752134

[B55] Magklara A. Lomvardas S. (2013). Stochastic gene expression in mammals: Lessons from olfaction. Trends Cell Biol. 23, 449–456. 10.1016/j.tcb.2013.04.005 23689023 PMC3755038

[B56] Margueron R. Reinberg D. (2010). Chromatin structure and the inheritance of epigenetic information. Nat. Rev. Genet. 11, 285–296. 10.1038/nrg2752 20300089 PMC3760772

[B57] Matzke M. A. Mosher R. A. (2014). RNA-Directed DNA methylation: An epigenetic pathway of increasing complexity. Nat. Rev. Genet. 15, 394–408. 10.1038/nrg3683 24805120

[B58] Moffitt J. R. Lundberg E. Heyn H. (2022). The emerging landscape of spatial profiling technologies. Nat. Rev. Genet. 23, 741–759. 10.1038/s41576-022-00515-3 35859028

[B59] Murphy T. Thuret S. (2015). The systemic milieu as a mediator of dietary influence on stem cell function during ageing. Ageing Res. Rev. 19, 53–64. 10.1016/j.arr.2014.11.004 25481406

[B60] Nakamura M. Gao Y. Dominguez A. A. Qi L. S. (2021). CRISPR technologies for precise epigenome editing. Nat. Cell Biol. 23, 11–22. 10.1038/s41556-020-00620-7 33420494

[B61] Nanney D. L. (1958). Epigenetic control systems. Proc. Natl. Acad. Sci. U. S. A. 44, 712–717. 10.1073/pnas.44.7.712 16590265 PMC528649

[B62] Natoli G. Ostuni R. (2019). Adaptation and memory in immune responses. Nat. Immunol. 20, 783–792. 10.1038/s41590-019-0399-9 31213714

[B63] Neufer P. D. Bamman M. M. Muoio D. M. Bouchard C. Cooper D. M. Goodpaster B. H. (2015). Understanding the cellular and molecular mechanisms of physical activity-induced health benefits. Cell Metab. 22, 4–11. 10.1016/j.cmet.2015.05.011 26073496

[B64] Ninov N. Yun M. H. (2015). Current advances in tissue repair and regeneration: The future is bright. Regen. (Oxf) 2, 84–91. 10.1002/reg2.30 PMC4895311 27499870

[B65] Ocampo A. Reddy P. Martinez-Redondo P. Platero-Luengo A. Hatanaka F. Hishida T. (2016). *In vivo* amelioration of age-associated hallmarks by partial reprogramming. Cell 167, 1719–1733. 10.1016/j.cell.2016.11.052 27984723 PMC5679279

[B66] Pisco A. O. d’Hérouël A. F. Huang S. (2016). Conceptual confusion: The case of epigenetics. bioRxiv. 10.1101/053009

[B67] Post Y. Clevers H. (2019). Defining adult stem cell function at its simplest: The ability to replace lost cells through mitosis. Cell Stem Cell 25, 174–183. 10.1016/j.stem.2019.07.002 31374197

[B68] Ptashne M. (2007). On the use of the word 'epigenetic. Curr. Biol. 17, R233–R236. 10.1016/j.cub.2007.02.030 17407749

[B69] Rana B. K. Bourne P. E. Insel P. A. (2012). Receptor databases and computational websites for ligand binding. Methods Mol. Biol. 897, 1–13. 10.1007/978-1-61779-909-9_1 22674158

[B70] Rebo J. Mehdipour M. Gathwala R. Causey K. Liu Y. Conboy M. J. (2016). A single heterochronic blood exchange reveals rapid inhibition of multiple tissues by old blood. Nat. Commun. 7, 13363. 10.1038/ncomms13363 27874859 PMC5121415

[B71] Ren R. Ocampo A. Liu G. H. Izpisua Belmonte J. C. (2017). Regulation of stem cell aging by metabolism and epigenetics. Cell Metab. 26, 460–474. 10.1016/j.cmet.2017.07.019 28826795

[B72] Robbins P. D. Jurk D. Khosla S. Kirkland J. L. LeBrasseur N. K. Miller J. D. (2021). Senolytic drugs: Reducing senescent cell viability to extend health span. Annu. Rev. Pharmacol. Toxicol. 61, 779–803. 10.1146/annurev-pharmtox-050120-105018 32997601 PMC7790861

[B73] Rumienczyk I. Kulecka M. Ostrowski J. Mar D. Bomsztyk K. Standage S. W. (2021). Multi-organ transcriptome dynamics in a mouse model of cecal ligation and puncture-induced polymicrobial sepsis. J. Inflamm. Res. 14, 2377–2388. 10.2147/JIR.S307305 34113146 PMC8184233

[B74] Russo V. E. A. Martienssen R. A. Riggs A. D. (1996). Epigenetic mechanisms of gene regulation. Cold Spring Harbor Laboratory Press.

[B75] Schaum N. Lehallier B. Hahn O. Palovics R. Hosseinzadeh S. Lee S. E. (2020). Ageing hallmarks exhibit organ-specific temporal signatures. Nature 583, 596–602. 10.1038/s41586-020-2499-y 32669715 PMC7757734

[B76] Seldin M. M. Koplev S. Rajbhandari P. Vergnes L. Rosenberg G. M. Meng Y. (2018). A strategy for discovery of endocrine interactions with application to whole-body metabolism. Cell Metab. 27, 1138–1155. 10.1016/j.cmet.2018.03.015 29719227 PMC5935137

[B77] Sender R. Milo R. (2021). The distribution of cellular turnover in the human body. Nat. Med. 27, 45–48. 10.1038/s41591-020-01182-9 33432173

[B78] Singh K. K. (2021). Mitochondrial secrets of youthfulness. Plast. Reconstr. Surg. 147, 33S–37S. 10.1097/PRS.0000000000007619 33347072

[B79] Singh P. P. Demmitt B. A. Nath R. D. Brunet A. (2019). The genetics of aging: A vertebrate perspective. Cell 177, 200–220. 10.1016/j.cell.2019.02.038 30901541 PMC7592626

[B80] Sorriento D. Di Vaia E. Iaccarino G. (2021). Physical exercise: A novel tool to protect mitochondrial health. Front. Physiol. 12, 660068. 10.3389/fphys.2021.660068 33986694 PMC8110831

[B81] Strahl B. D. Allis C. D. (2000). The language of covalent histone modifications. Nature 403, 41–45. 10.1038/47412 10638745

[B82] Stuart T. Satija R. (2019). Integrative single-cell analysis. Nat. Rev. Genet. 20, 257–272. 10.1038/s41576-019-0093-7 30696980

[B83] Tabula Sapiens C. Jones R. C. Karkanias J. Krasnow M. A. Pisco A. O. Quake S. R. (2022). The tabula Sapiens: A multiple-organ, single-cell transcriptomic atlas of humans. Science 376, eabl4896. 10.1126/science.abl4896 35549404 PMC9812260

[B84] Takahashi K. Yamanaka S. (2006). Induction of pluripotent stem cells from mouse embryonic and adult fibroblast cultures by defined factors. Cell 126, 663–676. 10.1016/j.cell.2006.07.024 16904174

[B85] Takamiya S. Shiotani K. Ohnuki T. Osako Y. Tanisumi Y. Yuki S. (2021). Hippocampal CA1 neurons represent positive feedback during the learning process of an associative memory task. Front. Syst. Neurosci. 15, 718619. 10.3389/fnsys.2021.718619 34552474 PMC8450371

[B86] Tan Y. Wei Z. Chen J. An J. Li M. Zhou L. (2019). Save your gut save your age: The role of the microbiome in stem cell ageing. J. Cell Mol. Med. 23, 4866–4875. 10.1111/jcmm.14373 31207055 PMC6653314

[B87] Tarasoff-Conway J. M. Carare R. O. Osorio R. S. Glodzik L. Butler T. Fieremans E. (2015). Clearance systems in the brain-implications for Alzheimer disease. Nat. Rev. Neurol. 11, 457–470. 10.1038/nrneurol.2015.119 26195256 PMC4694579

[B88] Tetta C. Bellomo R. Ronco C. (2003). Artificial organ treatment for multiple organ failure, acute renal failure, and sepsis: Recent new trends. Artif. Organs 27, 202–213. 10.1046/j.1525-1594.2003.00963.x 12662203

[B89] Tian X. J. Zhang H. Sannerud J. Xing J. (2016). Achieving diverse and monoallelic olfactory receptor selection through dual-objective optimization design. Proc. Natl. Acad. Sci. U. S. A. 113, E2889–E2898. 10.1073/pnas.1601722113 27162367 PMC4889386

[B90] Tierney M. T. Stec M. J. Rulands S. Simons B. D. Sacco A. (2018). Muscle stem cells exhibit distinct clonal dynamics in response to tissue repair and homeostatic aging. Cell Stem Cell 22, 119–127. 10.1016/j.stem.2017.11.009 29249462 PMC5945549

[B91] Ung C. Y. Weiskittel T. M. Correia C. Kaufmann S. H. Li H. (2022). Manifold medicine: A schema that expands treatment dimensionality. Drug Discov. Today 27, 8–16. 10.1016/j.drudis.2021.09.016 34600126 PMC8714694

[B92] Villeda S. A. Luo J. Mosher K. I. Zou B. Britschgi M. Bieri G. (2011). The ageing systemic milieu negatively regulates neurogenesis and cognitive function. Nature 477, 90–94. 10.1038/nature10357 21886162 PMC3170097

[B93] Villeda S. A. Plambeck K. E. Middeldorp J. Castellano J. M. Mosher K. I. Luo J. (2014). Young blood reverses age-related impairments in cognitive function and synaptic plasticity in mice. Nat. Med. 20, 659–663. 10.1038/nm.3569 24793238 PMC4224436

[B94] Waddington C. H. (1942). Canalization of development and the inheritance of acquired characters. Nature 150, 563–565. 10.1038/150563a0 13666847

[B95] Waddington C. H. (1942). The epigenotype. Int. J. Epidemiol. 41, 10–13. 10.1093/ije/dyr184 22186258

[B96] Wang S. W. Fan L. (1990). Clinical features of multiple organ failure in the elderly. Chin. Med. J. Engl. 103, 763–767. 2123779

[B97] Wei M. Brandhorst S. Shelehchi M. Mirzaei H. Cheng C. W. Budniak J. (2017). Fasting-mimicking diet and markers/risk factors for aging, diabetes, cancer, and cardiovascular disease. Sci. Transl. Med. 9, eaai8700. 10.1126/scitranslmed.aai8700 28202779 PMC6816332

[B98] Wolkenhauer O. Auffray C. Brass O. Clairambault J. Deutsch A. Drasdo D. (2014). Enabling multiscale modeling in systems medicine. Genome Med. 6, 21. 10.1186/gm538 25031615 PMC4062045

[B99] Xie L. Kang H. Xu Q. Chen M. J. Liao Y. Thiyagarajan M. (2013). Sleep drives metabolite clearance from the adult brain. Science 342, 373–377. 10.1126/science.1241224 24136970 PMC3880190

[B100] Xiong W. Ferrell J. E. Jr. (2003). A positive-feedback-based bistable 'memory module' that governs a cell fate decision. Nature 426, 460–465. 10.1038/nature02089 14647386

[B101] Zheng H. Xie W. (2019). The role of 3D genome organization in development and cell differentiation. Nat. Rev. Mol. Cell Biol. 20, 535–550. 10.1038/s41580-019-0132-4 31197269

